# Rapid estimation of nutritional elements on citrus leaves by near infrared reflectance spectroscopy

**DOI:** 10.3389/fpls.2015.00571

**Published:** 2015-07-23

**Authors:** Luis Galvez-Sola, Francisco García-Sánchez, Juan G. Pérez-Pérez, Vicente Gimeno, Josefa M. Navarro, Raul Moral, Juan J. Martínez-Nicolás, Manuel Nieves

**Affiliations:** ^1^Departamento de Producción Vegetal y Microbiología, Miguel Hernández UniversityAlicante, Spain; ^2^Departamento de Nutrición Vegetal, Centro de Edafología y Biología Aplicada del Segura, Consejo Superior de Investigaciones CientíficasMurcia, Spain; ^3^Unidad Asociada de Fertirriego y Calidad Hortofrutícola (Instituto Murciano de Investigación y Desarrollo Agrario y Alimentario – Centro de Edafología y Biología Aplicada del Segura), MurciaSpain; ^4^Departamento de Citricultura, Instituto Murciano de Investigación y Desarrollo Agrario y AlimentarioMurcia, Spain; ^5^Departamento de Agronomia, Universidad ISASantiago De Los Caballeros, Republica Dominicana

**Keywords:** calibration, macronutrients, micronutrients, NIRS, nitrogen, nutritional status

## Abstract

Sufficient nutrient application is one of the most important factors in producing quality citrus fruits. One of the main guides in planning citrus fertilizer programs is by directly monitoring the plant nutrient content. However, this requires analysis of a large number of leaf samples using expensive and time-consuming chemical techniques. Over the last 5 years, it has been demonstrated that it is possible to quantitatively estimate certain nutritional elements in citrus leaves by using the spectral reflectance values, obtained by using near infrared reflectance spectroscopy (NIRS). This technique is rapid, non-destructive, cost-effective and environmentally friendly. Therefore, the estimation of macro and micronutrients in citrus leaves by this method would be beneficial in identifying the mineral status of the trees. However, to be used effectively NIRS must be evaluated against the standard techniques across different cultivars. In this study, NIRS spectral analysis, and subsequent nutrient estimations for N, K, Ca, Mg, B, Fe, Cu, Mn, and Zn concentration, were performed using 217 leaf samples from different citrus trees species. Partial least square regression and different pre-processing signal treatments were used to generate the best estimation against the current best practice techniques. It was verified a high proficiency in the estimation of N (Rv = 0.99) and Ca (Rv = 0.98) as well as achieving acceptable estimation for K, Mg, Fe, and Zn. However, no successful calibrations were obtained for the estimation of B, Cu, and Mn.

## Introduction

Spain is the largest exporter of fresh fruit in Europe, with more than 50% of production being commercialized abroad. Citrus is one of most important crops in Spain, with 330,000 hectares currently dedicated to its production along the Mediterranean coast, producing 6.3 million tons of fruit annually. Sweet orange is the most common crop representing 48% of production, with mandarins and lemons accounting for 35% and 16% of production respectively. Like other fruit trees, citrus cultivation requires the right balance of nitrogen, potassium, phosphorous, and trace elements like manganese, boron, copper, and magnesium for vigorous growth and maximum fruit production. Conventionally, leaf analysis has provided a guide for fertilizer applications, according to the sufficiency range (SR), a method based on constructing independent nutrient indices, and including only one nutrient in each index (Walsh, 1973; Jones et al., 1991). A second method is based on dependent nutrient indices, in which each index includes two or more nutrients. Diagnosis and Recommendation Integrated System (DRIS) is the principal example of this approach (Beaufils, 1973).A good fertilization program must pay attention to how the plant mineral status changes through the phenological stages; adapting fertilizer application to meet the requirements of the trees during each stage. Monitoring the effect of plant nutrition on fruit development can require a number of complex chemical analyses in the laboratory, with some experiments needing to run over several years in order to draw the relevant conclusions. This often results in a large number of samples that must be analyzed; which is very time-consuming work, leading to high economic costs and, obviously, have a negative environmental impact owing to the production of noxious chemicals during the analysis techniques. Therefore, the development of a fast, environmentally friendly and cheaper method of analysis would be highly desirable.

Near infrared reflectance spectroscopy (NIRS) has the potential to be a useful tool in the quick analysis of numerous samples collected from long term experiments. The main benefits of using this technique are that – after calibration for the element of interest – it is possible to obtain an accurate quantitative estimation for the element in about a minute, without the use of chemical reagents and therefore, without producing pollutants. Additionally, this technique is less expensive than the traditional chemical based techniques and does not require a laborious preparation of samples before analysis.

Near infrared reflectance spectroscopy is based on the absorption of energy by various bonds, such as C–H, C–C, C = C, C–N, and O–H, which are characteristic of organic matter (Ludwig and Khanna, 2001), in the near infrared spectral range (700–2,500 nm). In addition, the mineral composition of an organic matrix can be estimated by NIRS owing to the association between minerals and organic functional groups or the organic matrix itself (Huang et al., 2008). So, samples with different organic composition will have a different near-infrared spectrum. However, in order to accurately estimate the concentration of certain elements in a sample using NIRS, the spectral analysis must first be calibrated against the absorption of known-concentration samples for the element of interest. Besides, the spectrum used in the calibration step must be similar, regarding the organic composition, between them.

There are numerous studies that demonstrate the capability of the NIRS technique concerning the estimation of different elements in plant species for a variety of different purposes. For example, NIRS technology has been successfully used to predict the nutritional quality of forage samples (Alomar et al., 2003), the mineral concentration in alfalfa (Halgerson et al., 2004), the quantification of nitrogen concentration in perennial ryegrass and red fescue (Gislum et al., 2004), and for the quality assessment of tomato landraces (García-Martínez et al., 2012). Additionally, there are studies that have demonstrated the usefulness and accuracy of the NIRS in isolation, or in combination with the visible spectroscopy (Vis/NIRS), as a predictive tool for the analysis of citrus products. Examples of this include the analysis of acidity, soluble solids and firmness in mandarins (Hernández Gómez et al., 2006), the measurement of the soluble solids content in oranges (Cayuela, 2008) and citrus fruits before harvest (Zude et al., 2008) and in the classification and analysis of citrus oils (Steuer et al., 2001).

Some works on the analysis of citrus leaf can be found, showing that through the combination Vis/NIRS, it is possible to obtain quantitative estimations of several elements in orange tree leaves (Min et al., 2008; Menesatti et al., 2010). However, there is an absence of studies on the use of the NIRS on leaves across different citrus species. The broad scale applicability of this technique for the quantitative estimation of macro and micronutrients must be demonstrated before it can be considered as a viable alternative to laboratory based techniques. Therefore, the aim of this study was to explore the predictive ability of the NIRS in the evaluation of several elements in citrus leaves of different species.

## Materials and Methods

### Citrus Leaves Samples

A total of 217 leaf samples from different species were used in this research, including 112 of ‘Verna’ lemon (*Citrus limon* Burm. F.), 21 ‘Carrizo’ citrange (*Citrus sinensis* × *Poncirus trifoliata*), 21 sour orange (*C. aurantium* L.), 21 *C. macrophylla* (*C. macrophylla* Wester), 15 ‘Clemenules’ mandarin (*C. reticulata* Blanco), 15 ‘Lane late’ navel orange (*C. sinensis* L. Osb.) and 12 ‘Star Ruby’ grapefruit (*C. paradise* Macf.). The six species studied in this experiment were collected from the citrus collection available from the CEBAS–CSIC experimental farm ‘Trescaminos’ in Santomera (Murcia, Spain) and IMIDA experimental orchard in Torrepacheco (Murcia, Spain).

### Analytical Methods

The leaves were briefly rinsed with deionised water, oven-dried at 60°C for at least 48 h, and ground to a fine powder. Scanning a ground sample by NIRS can improve the homogeneity of the sample and obtaining repetitive spectra. The mineral concentrations were determined by inductively coupled plasma emission optical spectrometry (Iris Intrepid II, Thermo Electron Corporation, Franklin, MA, USA) in a 0.1 g sample after an acid digestion in HNO_3_:H_2_O_2_ (5:3 by volume) in a microwave that reached 190°C in 20 min and held at this temperature for 2 h (CEM Mars Xpress, Matthews, NC, USA). The nitrogen concentration was determined using a Thermo- Finnigan 1112 EA elemental analyser (Thermo-Finnigan, Milan, Italy).

### NIRS Analysis

Near infrared reflectance spectroscopy analysis was performed using a FT-NIR spectrometer (MPA, Bruker Optik GmbH, Germany) in the wave range 12000 –3800 cm-1 (830–2600 nm) with steps of 8 cm-1. Each ground sample was placed in a rotating glass plate of 12 cm in diameter (similar to the Petri dishes), scanned three times using Opus software (version 6, ^©^Bruker Optik), recording absorbance, as log 1/R, where R is reflectance, for a total of 64 scans per sample. The three spectra of each sample were averaged. Due to the rotation of the plate, it was possible to take signal data from different points of the sample. The glass plate must be fully covered with the ground sample. The resulting layer should be at least half a cm thick. Normally, 20–25 g of sample are enough.

**FIGURE 1:**
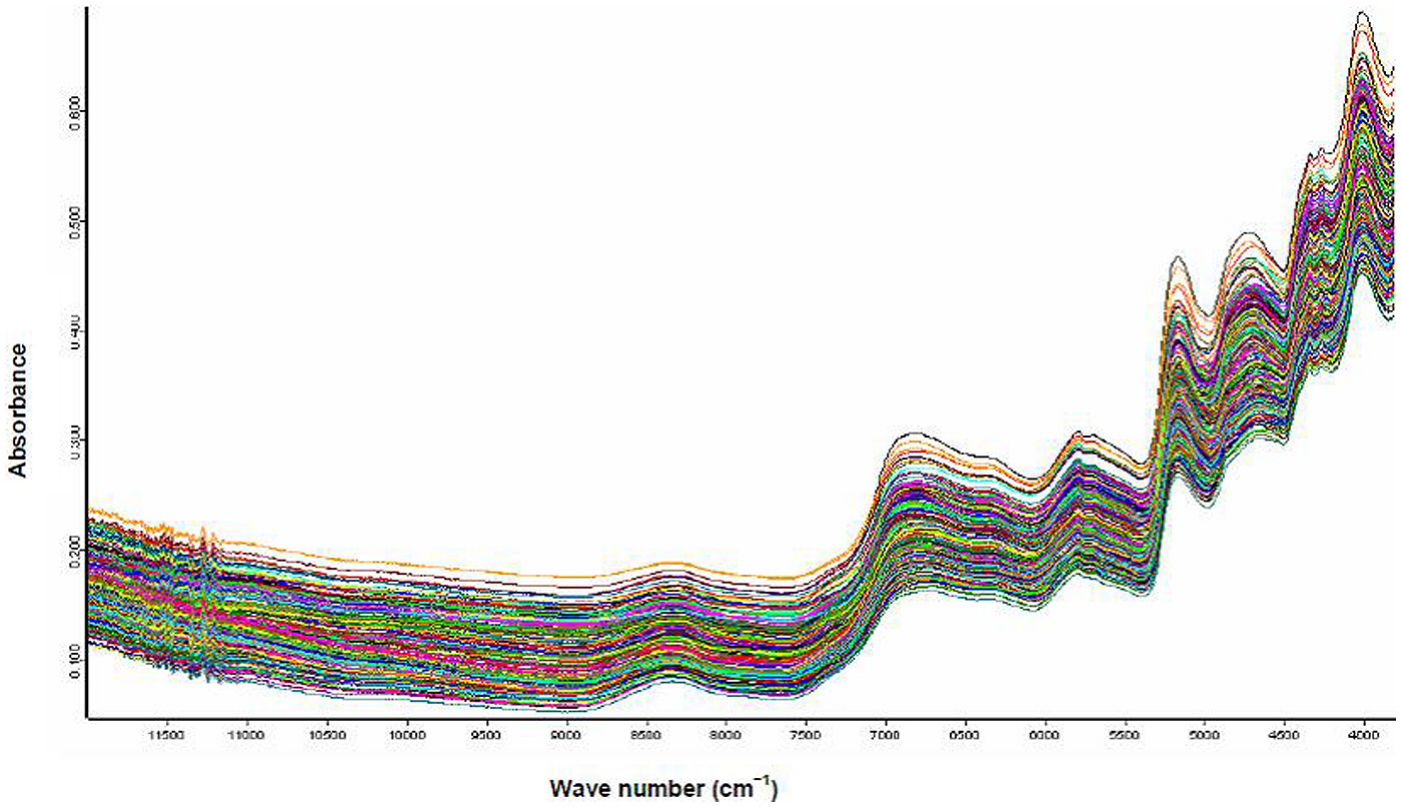
**Typical log (1/R) spectra for dry ground citrus leaves samples**.

**Figure 1** shows the NIRS spectra of the citrus leaves samples.

The set of samples mentioned in Section “Citrus Leaves Samples” was divided into two parts: one of 175 samples used for the calibration step (calibration set) and the remaining 42 samples (∼20% of the total set) used for the external validation step (validation set). Within the validation set, samples were selected to keep as much similarity from original sample as possible, however, the resultant proportions of the seven citrus varieties varied. The validation set included the following samples: 20 ‘Verna’ lemon, 4 ‘Carrizo’ citrange, 4 sour orange, 3 *Citrus macrophylla*, 4 ‘Clemenules’ mandarin, 4 ‘Lane late’ navel orange and 3 ‘Star Ruby’ grapefruit. The sample set was split to create the validation set not used in the calibration, to allow for faster processing without the internal validation (cross validation) required when dealing with a large number of samples.

Pre-treatment of spectral data was important to fully or partly eliminate the systematic errors that could be caused by various factors (Galvez-Sola et al., 2010). The following methods were applied: vector normalisation (VN), minimum–maximum normalisation (MMN), multiplicative scatter correction (MSC), first derivative (FD), second derivative (SED), straight line subtraction (SLS) and linear offset subtraction (LOS). A brief explanation of these pre-processing methods can be found in Galvez-Sola et al. (2013). Partial least square regression (PLSR) was used throughout the calibration process, to ensure a good correlation between the spectral data and the concentration values, while different spectra pre-processing methods were tested.

No general recommendation can be given whether the data set should be pre-processed or which method would be best suited. Therefore, the optimal data pre-processing method can only be found empirically by applying several methods in isolation or as a combination to the spectral data, and comparing the results.

To evaluate the estimation, several statistical parameters were performed:

– *R*_c_: coefficient of determination for calibration.
– RMSEE: root mean square error of estimation (calibration step).
– RPD: calculated as the standard deviation divided by the standard error of prediction. (The higher this value, the better)
– *F*: number of factors or principal components.
– *R*_v_: coefficient of determination for validation.
– RMSEP: root mean square error of prediction (validation step).
– Bias: is the difference between the mean real value and the mean estimated value for the validation set samples (validation step).

Malley et al. (2004) suggest a guideline scale for describing the performance of calibrations for environmental samples: Excellent *R*_v_ > 0.95, RPD > 4; Successful, *R*_v_ = 0.9–0.95, RPD 3–4; Moderately Successful, *R*_v_ = 0.8–0.9, RPD 2.25–3; and Moderately Useful, *R*_v_ = 0.7-0.8, RPD 1.75-2.25. This guideline was used to evaluate the calibrations in this experiment.

### Nutritional Diagnostic Indices

Leaf nutrient analysis from 112 samples ‘Verna’ lemon plants, mentioned above, were interpreted according to DRIS (Beaufils, 1973). They only provided a basis for comparing the sufficiency of each element relative to other elements, with a high-yield population providing the standard for comparison. For the DRIS index, the mean nutrient ratios used were selected from each pair of inversely related ratios (P: K, K: P) showing the lowest SD. Reference DRIS norms for Verna lemon leaves were used to calculate DRIS index (Cerdá et al., 1995). These DRIS index were used to evaluate the influence of both analytical methods and NIRS estimation on that.

## Results and Discussion

**Table 1** shows the mean values, concentration ranges and standard deviation for each element identified from the analytical characterisation of the citrus leaves by classical techniques. This data was used as the ‘true’ element concentration which was used to evaluate the accuracy of the model estimations from the spectral data. The model was considered to be optimized when the estimated value for the element of interest lay between the same range values.

**Table 1:** Range and mean values of the studied elements in the 217 citrus leaves samples, expressed on dry matter basis.

Elements	Range	Mean	SD^a^
N (g 100 g^-1^)	1.07–3.86	2.48	0.67
K (g 100 g^-1^)	0.42–1.92	1.13	0.35
Ca (g 100 g^-1^)	0.89–6.77	3.54	1.59
Mg (g 100 g^-1^)	0.14–0.80	0.33	0.13
B (mg kg^-1^)	24.22–509.93	110.19	84.84
Fe (mg kg^-1^)	39.54–636.75	121.51	96.01
Cu (mg kg^-1^)	0.83–11.24	4.59	1.52
Mn (mg kg^-1^)	18.69–70.48	38.54	9.68
Zn (mg kg^-1^)	9.01–43.92	19.27	8.04

*^a^SD, standard deviation.*

**Table 2** shows the leaf macro and micro-nutrients concentration for the seven plant species used in this experiment. In all nutrients analyzed it was observed significant differences among species, except for leaf Cu concentration. In addition, **Table 1** also show that leaf mineral concentrations ranged in wide intervals. Therefore, these data reflected that using these seven trees species we had a wide range of leaf mineral concentrations. It is important for determining if NIR is a useful technique to predict nutritional status of crops with different requirements.

**Table 2:** Leaf macro and micro-nutrient concentration in the seven citrus plant species.

	Leaf macronutrient concentration (g 100 g^-1^ dw)				
Plant species	N	K	Ca	Mg	
Carrizo citrange	3.59 a	1.53 a	1.87 c	0.35 b	
Clemenules mandarin	2.69 c	0.56 c	5.99 a	0.56 a	
Lane late orange	2.82 c	0.69 c	5.75 a	0.49 a	
Verna lemon	1.93 d	1.16 b	3.75 b	0.27 bc	
Citrus Macrophylla	3.30 ab	1.42 ab	1.25 c	0.17 c	
Sour orange	2.95 bc	1.35 ab	1.37 c	0.30 b	
Star ruby grapefruit	2.61 c	0.58 c	5.77 a	0.59 a	

	ANOVA			
	^∗∗∗^	^∗∗∗^	^∗∗∗^	^∗∗∗^	

	**Leaf micronutrient concentration (mg kg^-1^ dw)**				
	**B**	**Fe**	**Cu**	**Mn**	**Zn**

Carrizo citrange	96.87 b	237.03 ab	5.59	48.65 a	26.61 b
Clemenules mandarin	206.98 a	75.31 c	2.64	43.86 ab	32.39 ab
Lane late orange	126.91 ab	74.15 c	3.69	40.71 abc	36.78 a
Verna lemon	79.76 b	95.55 bc	4.69	35.85 bc	14.51 c
Citrus Macrophylla	44.97 b	106.22 bc	6.02	29.42 c	15.99 c
Sour orange	76.79 b	345.07 a	6.20	39.60 abc	13.97 c
Star ruby grapefruit	198.71 a	65.83 c	4.11	43.06 ab	31.23 ab

	ANOVA				
	^∗∗^	^∗∗^	ns	^∗∗^	^∗∗^

*Inside of each column, means (*n* = 4) with the same letter are not significantly different from each other (Duncan test *p* > 0.05). ns indicate non-significant effects; ^∗^, ^∗∗^ and ^∗∗∗^ indicates significant differences at *p* < 0.05, 0.01, and 0.001, respectively.*

**FIGURE 2:**
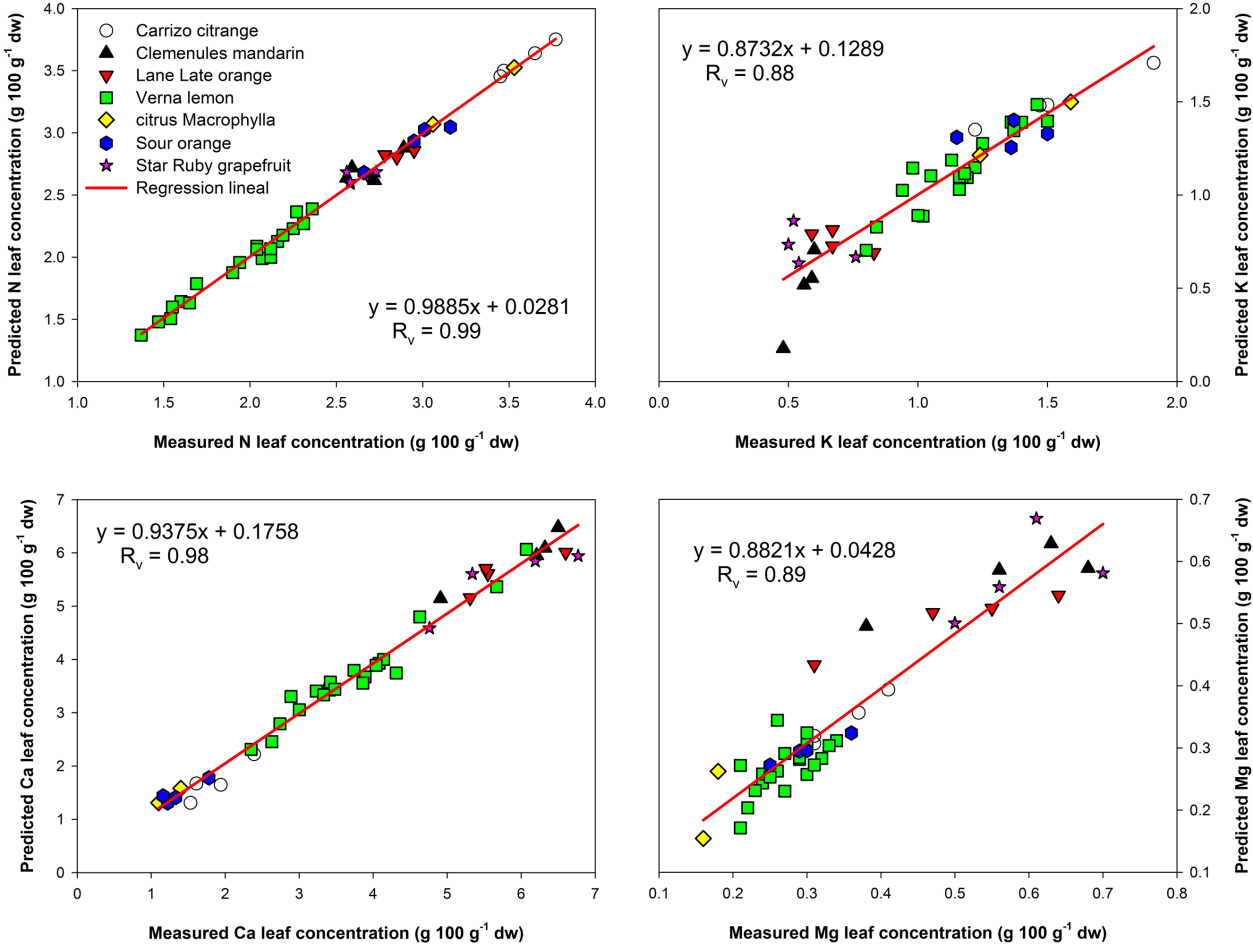
**Near infrared reflectance spectroscopy validation plots between predicted and measured values for N, K, Ca, and Mg**.

The N estimation model was the best predictive model obtained in this study, with very good results in both the calibration and validation processes. The errors in estimation were very low and the coefficient of determination for the validation step was 0.99 (**Table 3**). In addition, the high RPD obtained supported the results and confirmed the high accuracy of this calibration, giving it a ranking of ‘Excellent’ in the performance scale (Malley et al., 2004). **Figure 2** shows the calibration plot for this element.

**Table 3:** Near infrared reflectance spectroscopy calibration and validation results for all studied elements.

Elements	Calibration^a^			Validation^b^				Factors	Spectrum region (cm^-1^)	Preprocessing^c^
	*R*_c_	RMSEE	RPD	*R*_v_	RMSEP	RPD	Bias			
N (g 100 g^-1^)	0.98	0.09	7.89	0.99	0.06	10.9	0.0004	11	6102-4598	MMN
K (g 100 g^-1^)	0.94	0.09	3.95	0.88	0.12	2.86	0.0063	17	7502-4598	FD + VN
Ca (g 100 g^-1^)	0.98	0.25	6.39	0.98	0.25	6.94	0.0627	13	7502-4598	FD
Mg (g 100 g^-1^)	0.84	0.05	2.52	0.89	0.05	2.97	-0.0007	8	7502-4248	MMN
B (mg kg^-1^)	0.56	59.00	1.51	0.47	53.20	1.38	0.0521	6	11996-7498	MSC
Fe (mg kg^-1^)	0.77	44.00	2.1	0.76	60.40	2.04	-3.23	15	7502-5446	MMN
Cu (mg kg^-1^)	0.22	1.29	1.13	0.36	1.47	1.22	0.12	4	5450-4248	MMN
Mn (mg kg^-1^)	0.77	4.99	2.08	0.53	5.55	1.46	-0.394	12	11996-6098	—
Zn (mg kg^-1^)	0.84	3.23	2.49	0.88	3.34	2.84	0.0683	17	7502-5446	VN

*^a^*R*_c_, coefficient of determination for calibration; RMSEE, root mean square error of estimation; RPD, calculated as the SD divided by the SE of prediction.*

*^b^*R*_v_, coefficient of determination for validation; RMSEP, root mean square error of prediction; Bias, difference between the mean real value and the mean estimated value for the validation set samples.*

*^c^Preprocessing: VN, vector normalization; MSC, multiplicative scatter correction; FD, first derivative; MMN, minimun–maximun normalization; —, no treatment.*

Satisfactory results were also obtained for the K calibration. In this case, *R*_v_ was smaller than that of the N model, but still good enough for carrying out a prediction for this macronutrient with low errors of estimation. The coefficient of determination in the calibration process was higher than for the validation: 0.94 and 0.88 respectively. Given this situation, it would be desirable to increase the number of samples of the data set and recalculate the model in order to obtain comparable results for both steps (Malley et al., 2004). However, these results still achieve a performance score of ‘Moderately Successful.’

The results obtained for Ca estimation showed that NIRS spectra can again be used as a good predictive model, with very good results in both the calibration and validation processes. *R*_c_ and *R*_v_ coefficients were 0.98 and the RPD obtained for both steps were high (**Table 3**), supporting the use of NIRS as a great prediction model for this element (**Figure 2**) with a performance score of ‘Excellent.’

The results obtained for Mg were also promising achieving a performance score of both coefficients *R*_c_ and *R*_v_ and the RPD were somewhat lower (compared with the Ca model), but the resulting model did not have a high root mean square errors of estimation, as can be seen in **Table 3** and in **Figure 2**. Therefore, the evidence is sufficient to support the use of NIRS as a predictor for this element. These findings support those of Menesatti et al. (2010) which also reported good calibrations for some elements, including N, K, Ca, and Mg, but using Vis-NIR spectroscopy on orange leaves.

In contrast, the estimations for B and Cu content were not adequate. In these cases the NIRS estimation method could not generate accurate values for the element concentration, with all statistical parameters far below what would be considered as acceptable for use in the field (**Table 3**).

The results for Fe estimation were near the limit of what would be considered acceptable. The coefficient *R*_v_ was 0.76 and the RPD slightly higher than 2 (**Table 3**).

**FIGURE 3:**
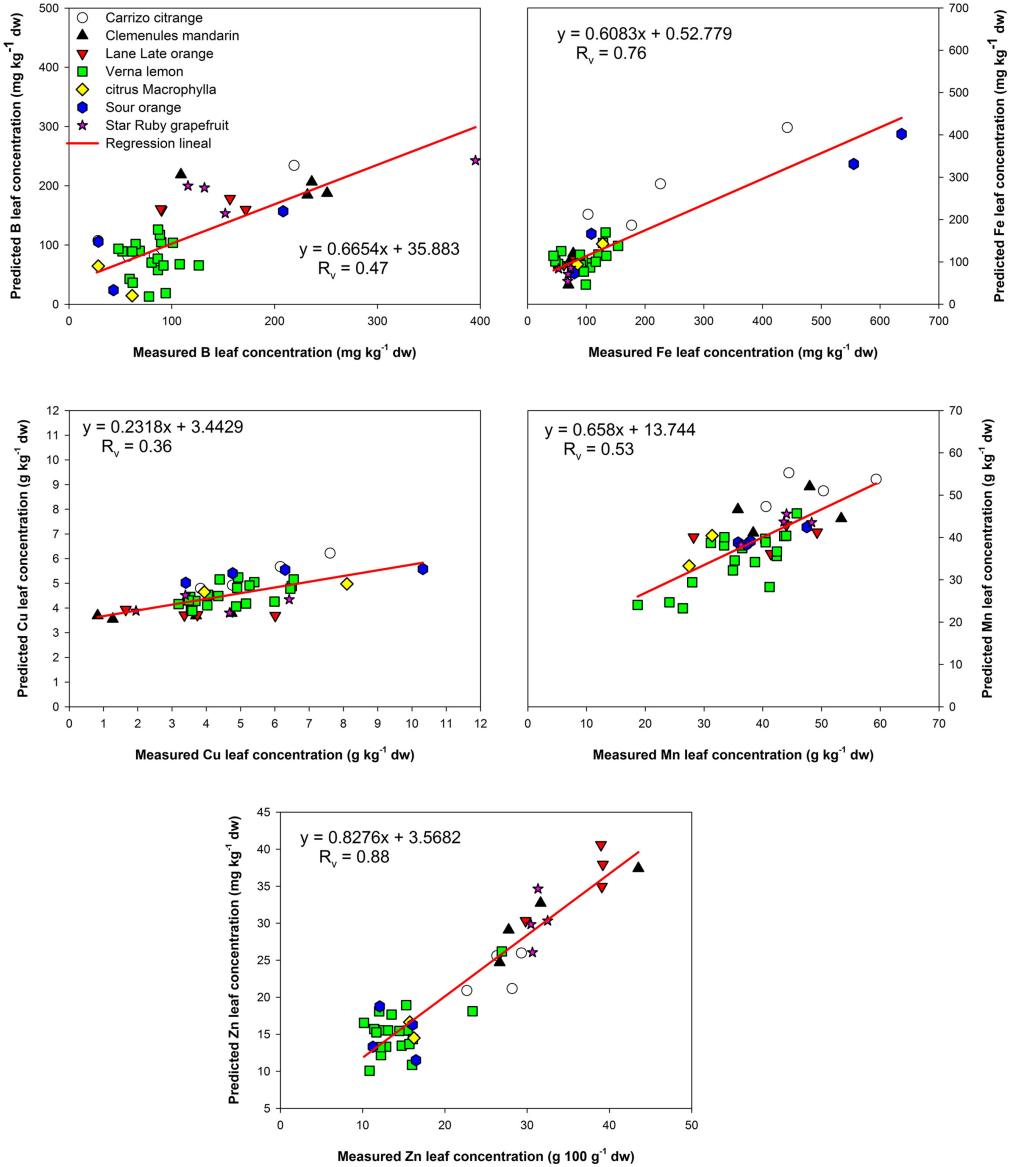
**Near infrared reflectance spectroscopy validation plots between predicted and real (measured) values for B, Fe, Cu, Mn, and Zn**.

Therefore, estimating this element by NIRS would require the acceptance of a prediction error, and this error would increase as the iron concentration of the unknown sample increased (**Figure 3**). The range of Fe concentrations within the set of samples was varied (**Table 1**) and skewed, with 90.3% of the samples having a value less than 200 mg kg^-1^, and with few samples exceeding this value. This could adversely affect the generation of the estimation model resulting in the lower coefficients observed.

Interestingly, in the case of Mn estimation, the calibration set results were acceptable, but the validation results did not support the calibration statistics. This indicated it would be beneficial to use more samples to generate another model, in order to achieve a better predictive result, where the calibration and validation sets were analogous.

Finally, Zn content was also a good candidate for estimation by NIRS, achieving a performance score of ‘Moderately Successful.’ The predictive model resulted in a coefficient of determination validation of 0.88 and a RPD = 2.84 supporting the accuracy of the estimation.

The good calibration results for most of the studied elements support the evidence that NIRS can be used to accurately estimate the nutrient concentration of some elements in citrus leaves; and additionally, that this technique is applicable across citrus species. The spectra results between species were very similar, with the spectral differences observed not interfering with obtaining good calibrations for the elements of interest.

Furthermore, the number of factors (or principal components) was adequate within all calibrations. He et al. (2007), state that for all calibrations, the number of factors must be not higher than 1/10 the number of samples used in the calibration step. Thus, in this study, the ideal number of factors was 17 or less and this value was not exceeded in any case.

The spectral regions (shown in **Table 3**) highlight the areas where the spectral information was collected for each element calibration. Interestingly this region usually varied depending on the pre-processing method used. This suggests that further development of the pre-possessing methodology could yield improvements in the estimation accuracy for those elements that scored badly in the performance scale by targeting different areas of the spectra.

Leaf nutrient concentration varied from deficiency level to excess. DRIS index approach provides a basis for determining which element, if any, is likely to limit yield. Because each DRIS index measures deviations from a specific norm, all values in one foliar sample must add up to zero (within a round off error of plus or minus one). The analytical method did not affect the accuracy of the DRIS index since those add up to zero in each sample for both analytical methods (classical techniques and NIRS estimation). The average differences between the DRIS index from both analytical methods for N, K, Ca, and Mg were 3, 3, 5, and 11, respectively. The differences between DRIS index compared to results of calibration show similar consistencies. Researchers have used an in-balance range as wide as -15 to +15 (Kelling and Schulte, 1986). A DRIS index less than -25 indicates a likely deficiency. Values greater than +100 may be an indication of possible nutrient excess. SR and DRIS approach identified nutrient excess and deficiencies in the same foliar samples. These differences between both analytical methods were similar to that observed between nutritional diagnostic indices, like DRIS and Plant Analysis with Standardized Scores, PASS (Baldock and Schulte, 1996).

In summary, according to the suggested guideline described in Section “NIRS Analysis,” the calibrations for N and Ca were ‘Excellent,’ those for K, Mg, and Zn were ‘Moderately Successful’ and the calibration for Fe was ‘Moderately Useful,’ with the NIRS calibrations for B, Cu, and Mn being inadequate.

## Conclusion

The results showed that NIRS can constitute a feasible technique to quantify several macro and micronutrients such as, N, K, Ca, Mg, Fe, and Zn in citrus leaves of different species. Therefore, NIRS would be a promising alternative to acquire a predictive view of the nutrient concentration of citrus leaves, thereby, facilitating the evaluation of the plant nutritional status of the trees. This technique provides a very interesting opportunity for future monitoring experiments, providing reliable results that can be obtained quickly, easily, and at low economic cost.

## Author Contributions

MN developed the research hypothesis and the study design. JP-P, VG, JN performed field sampling and chemical analysis. LG-S, FG-S performed NIR scanning, calibration model development. RM, JM-N performed statistical analysis. The final manuscript is the end product of joint writing efforts of all authors.

## Conflict of Interest Statement

The authors declare that the research was conducted in the absence of any commercial or financial relationships that could be construed as a potential conflict of interest.
